# Development of low blood glucose readings in nine non-diabetic patients treated with tumor necrosis factor-alpha inhibitors: a case series

**DOI:** 10.1186/1752-1947-6-5

**Published:** 2012-01-10

**Authors:** Jadwiga B Czajkowska, Brandon Shutty, Susan Zito

**Affiliations:** 1 Department of Rheumatology, Largo Medical Center, Nova Southeastern University College of Osteopathic Medicine, 2025 Indian Rocks Road S, Largo, FL 33774, USA; 2 Lake Erie College of Osteopathic Medicine, Bradenton, FL, USA

## Abstract

**Introduction:**

Treatment with various biological agents in disease states such as rheumatoid arthritis has been associated with multiple side effects. Whereas many of these are frequently reported in the literature, hypoglycemia, a possible side effect of tumor necrosis factor-alpha inhibitors, may be underpublicized.

**Case presentation:**

We report nine cases of non-diabetic Caucasian women who were between 29 and 68 years of age and who developed low glucose readings after treatment with tumor necrosis factor-alpha inhibitors. We provide a more detailed discussion of existing evidence of the role of tumor necrosis factor-alpha in the pathogenesis of inflammation and its impact on glycemic equilibrium.

**Conclusions:**

Physicians using tumor necrosis factor-alpha inhibitors in the treatment of various rheumatic and other autoimmune diseases should be aware of the potential for the development of glycemic disturbance in these patients. A further role of tumor necrosis factor-alpha inhibitors in the glycemic equilibrium warrants larger controlled trials in patients with and those without a history of diabetes.

## Introduction

Treatment with a biologic agent is useful for controlling inflammatory disease states, such as rheumatoid arthritis (RA), when disease-modifying anti-rheumatic drugs (DMARDs) are not enough to fully control their activities [1]. Tumor necrosis factor-alpha (TNF-α) is categorized as a pro-inflammatory cytokine. It is thought that this molecule is crucial in the initiation and continuation of inflammation in many rheumatic diseases, including RA, psoriatic arthritis, ankylosing spondylitis, and many others. Currently available TNF-α inhibitors (etanercept, infliximab, adalimumab, certolizumab pegol, and golimumab) aim to block pro-inflammatory actions of this cytokine [2]. Their effectiveness to finally control disease activities in various rheumatic diseases has been proven in many randomized controlled studies [3-5].

However, TNF-α inhibitors entail a plethora of concerns associated with their use. Some of the potential side effects and complications include increased risk of infusion reactions, life-threatening and opportunistic infections (tuberculosis and fungal and other atypical infections), malignancy, and existing concerns associated with their use during pregnancy [2].

Interestingly, TNF-α inhibitors may also play a role in glycemic control since the TNF-α molecule is known to affect glucose homeostasis. Outcomes affecting glycemic control may be an underpublicized side effect in the literature. The evidence linking inflammation and diabetes mellitus (DM) dates back more than a century. Studies in mice showed a positive correlation between TNF-α quantity and insulin resistance [6]. Additionally, other studies have been confirmed in humans, in both those with and those without diabetes mellitus type II (DM II) [7]. Furthermore, insulin sensitivity was noted to improve in patients with prolonged infliximab treatment [8]. In this paper, we present nine patients who developed low glucose readings after treatment with TNF-α inhibitors.

### Case presentations

Table 1 includes detailed information regarding each of the nine patients presented in our paper.

**Table 1:** Descriptive characteristics of patients

Patient	Diagnosis	TNF-α inhibitor	DMARD	Age in years, BMI	Sex, race	Episodes of low glucose readings	Venous glucose value, mg/dL	Total duration on TNF-α inhibitor	Time before development of hypoglycemia	History of diabetes?	Family history of diabetes?	History of gestational diabetes?
1	RA	Infliximab, etanercept	Hydroxychloroquine	68, 22.0	Female, Caucasian	2	67, 68	13 months (infliximab), 10 months (abatacept)	6 months, 10 months	No	No	No
2	RA, SLE, CREST syndrome	Infliximab	Hydroxychloroquine, MTX	54, 32.4	Female, Caucasian	1	66	13 months (infliximab)	8 months	No	Yes	No
3	RA	Infliximab	Leflunomide	62, 22.1	Female, Caucasian	1	60	24 months (infliximab)	4 months	No	No	No
4	RA, SpA	Adalimumab	Hydroxychloroquine	29, 18.3	Female, Caucasian	1	67	3 months (adalimumab)	3 months	No	No	No
5	RA, FMS	Certolizumab, infliximab	Leflunomide	35, 19.3	Female, Caucasian	2	69, 63	11 months (abatacept), 14 months (infliximab)	2 months, 3 months	No	No	No
6	RA, SLE, CREST syndrome	Adalimumab, certolizumab, infliximab	Leflunomide	55, 24.5	Female, Caucasian	4	63, 62, 62, 68	17 months (adalimumab), 17 months (certolizumab)	4 months, 14 months, 15 months, 6 months	No	No	No
7	RA, vWD	Adalimumab, golimumab	MTX	30, 19.6	Female, Caucasian	3	68, 58	11 months (adalimumab), 3 months (golimumab)	6 months, 1 month	No	No	No
8	RA, SpA, FMS	Adalimumab	None	47, 21.4	Female, Caucasian	1	54	11 months (adalimumab)	2 months	No	No	No
9	RA, FMS	Infliximab	Hydroxychloroquine	65, 19.8	Female, Caucasian	1	64	24 months (infliximab)	5 months	No	No	No

BMI: body mass index; CREST: calcinosis, Raynaud syndrome, esophageal dysmotility, sclerodactyly, and telangiectasia; DMARD: disease-modifying anti-rheumatoid drug; FMS: fibromyalgia syndrome; MTX: methotrexate; RA: rheumatoid arthritis; SLE: systemic lupus erythematosus; SpA: spondyloarthropathy; TNF-α: tumor necrosis factor-alpha; vWD: von Willebrand disease.

In Case 1, a 68-year-old Caucasian woman with a history of RA was initially treated with hydroxychloroquine. Since her disease activity was not adequately controlled, additional treatment with TNF inhibitor infliximab at 3 mg/kg intravenously was initiated. She developed an episode of low blood glucose (a venous glucose level of 67 mg/dL) six months after starting treatment. Her infliximab was discontinued because of ineffectiveness, and treatment with another biologic agent, etanercept, at 50 mg subcutaneously weekly was started. Subsequently, she developed another episode of low blood glucose, of 68 mg/dL, 10 months after initiating treatment. So, overall, she developed two episodes of low blood glucose readings: one of 67 mg/dL and the other of 68 mg/dL. She was not symptomatic.

In Case 2, 54-year-old Caucasian woman had a history of RA, systemic lupus erythematosus (SLE), CREST (calcinosis, Raynaud syndrome, esophageal dysmotility, sclerodactyly, and telangiectasia) syndrome, and a family history of DM type II. She was treated with hydroxychloroquine and methotrexate. Owing to uncontrolled disease activity, TNF-α inhibitor infliximab was added. She developed one episode of low blood glucose reading of 66 mg/dL 8 months after starting treatment with infliximab. She was not symptomatic.

In Case 3, A 62-year-old Caucasian woman had a history of RA along with chronic anemia. Her RA was initially treated with leflunomide. Owing to uncontrolled disease activity, TNF-α inhibitor infliximab at 3 mg/kg intravenously was added to her treatment regimen. She developed one episode of low blood glucose reading of 60 mg/dL four months after starting infliximab. She was not symptomatic.

In Case 4, a 29-year-old Caucasian woman had a history of RA and seronegative spondyloarthropathy (SpA). She was treated with hydroxychloroquine, and then, owing to uncontrolled disease activity, TNF-α inhibitor adalimumab at 40 mg subcutaneously was added. She experienced one episode of low blood glucose reading with a value of 67 mg/dL three months after starting adalimumab. She was not symptomatic.

In case 5, a 45-year-old Caucasian woman with a history of RA and fibromyalgia syndrome (FMS) was initially treated with leflunomide. Subsequently, TNF-α inhibitor certolizumab at 200 mg subcutaneously every four weeks was added to her treatment regimen. She had an episode of low blood glucose reading with a value of 69 mg/dL two months after starting certolizumab. Owing to intolerance, she was switched to another TNF-α inhibitor, infliximab, at 3 mg/kg intravenously. After three months, another bout of low blood glucose reading, with a value of 63 mg/dL, occurred. So she developed two episodes of low blood glucose readings: one of 69 mg/dL after certolizumab and another, of 63 mg/dL, after infliximab. She was not symptomatic.

In case 6, a 55-year-old Caucasian woman with a history of RA, SLE, and CREST syndrome was treated with leflunomide at the beginning, and then after her disease activity was not controlled, TNF-α inhibitor adalimumab at 40 mg subcutaneously was added. She developed three episodes of low blood glucose readings of 63, 62, and 62 mg/dL four, 14, and 15 months, respectively, after starting adalimumab. Secondary to intolerance, another anti-TNF inhibitor, certolizumab, was started. Another episode of low blood glucose reading, of 68 mg/dL, developed six months after starting certolizumab. So she developed three low blood glucose readings of 63, 62, and 62 mg/dL, respectively, after initiation treatment with adalimumab, and then another episode of low blood glucose reading, of 68 mg/dL, after certolizumab. She was not symptomatic.

In case 7, a 30-year-old Caucasian woman with a history of von Willebrand disease and RA was initially treated with methotraxate. After uncontrolled disease activity, treatment with TNF-α inhibitor adalimumab at 40 mg subcutaneously was initiated. She developed one episode of low blood glucose reading with a value of 68 mg/dL six months after starting adalimumab. Secondary to intolerance, she was switched to golimumab at 50 mg subcutaneously. Then, one month after starting golimumab, another episode of low blood glucose reading was recorded at 58 mg/dL. So she had one episode of low blood glucose level of 68 mg/dL after adalimumab and then another episode of low blood glucose reading, of 58 mg/dL, after golimumab. She was not symptomatic.

In case 8, a 47-year-old Caucasian woman had a history of SpA, FMS, and RA. Since her disease activity was not well controlled, TNF-α inhibitor adalimumab at 40 mg subcutaneously every two weeks was initiated. Subsequently, patient developed one episode of low glucose reading of 54 mg/dL 2 months after starting adalimumab. She was not symptomatic.

In case 9, a 65-year-old Caucasian woman had a history of RA and FMS. Initially, she was treated with hydroxychloroquine. After uncontrolled disease activity, TNF-α inhibitor infliximab at 3 mg/kg intravenously was started. She developed an episode of low blood glucose level of 64 mg/dL five months after starting infliximab. She was not symptomatic.

## Discussion

Biologic agents in the category of TNF-α inhibitors, including etanercept, infliximab, adalimumab, certolizumab pegol, and golimumab, have proven efficacious in controlling disease activity of various rheumatic and autoimmune diseases, including RA, psoriatic arthritis, and ankylosing spondylitis, because of their action against the TNF-α molecule [9].

In this paper, nine patients treated with TNF-α inhibitors developed low blood glucose levels. Six of them were treated with infliximab, five with adalimumab, two with certolizumab pegol, and one with golimumab. The average time of developing hypoglycemia after initiating TNF-α inhibitor was 4.3 months; the shortest time was one month, and the longest time was six months. The level of hypoglycemia ranged from 54 to 69 mg/dL. All of the patients treated were Caucasian women ranging from 29 to 68 years of age. Only one patient treated had a family history of DM type II (case 2), but none of the patients had a personal history of DM type I or type II. All of the recordings of blood glucose levels were done retrospectively for approximately two hours in a post-prandial state.

The body mass index (BMI) in our patients ranged from 18.3 to 32.4, and the average was 22.15 (normal weight). One patient had a BMI of 18.3, placing her in the underweight category. Another patient had a BMI of 32.4, placing her in the obese category. The majority of the BMIs were within normal weight category and included the following values: 22.0, 22.1, 19.3, 24.5, 19.6, 21.4, and 19.8. It is known that higher BMI is associated with increased risk for developing diabetes type II along with cardiovascular disease such as coronary artery disease and high blood pressure. However, most of our patients who developed low blood glucose readings had BMIs within the normal weight category. This finding suggests that the impact of TNF-α inhibitors on glycemic equilibrium might be independent of BMI measurement.

While all nine patients had at least one bout of a low blood glucose reading, four patients (cases 1, 5, 6, and 7) developed more than one episode of hypoglycemia after initiating TNF-α inhibitor treatment. Case 1 was a patient who had RA and who developed one episode of a low blood glucose reading after treatment with infliximab and another low blood glucose reading after treatment with etanercept. Case 5 was a patient who had RA and who developed low blood glucose readings after treatment with certolizumab and then after treatment with infliximab. Case 5 was a patient who had RA and who developed a low blood glucose reading while being treated with certolizumab pegol and another low blood glucose reading after switching to infliximab. Case 6 was a patient who had RA, SLE, and CREST syndrome and who had the highest incidence of low blood glucose readings during this observational case series. Three episodes of low blood glucose readings occurred while she was on adalimumab, and one episode occurred while she was on certolizumab pegol. Case 7 had one episode of low blood glucose reading while she was on adalimumab and another episode while she was on golimumab.

The literature describes episodes of hypoglycemia occurring in those with pre-existing diabetes and concurrent use of TNF-α inhibitors. Boulton and Borne [10] report the case of a 55-year-old woman who had a history of type I diabetes and RA and who developed several episodes of hypoglycemia within three weeks after initiating etanercept and subsequently within a 24-hour period of starting adalimumab. Moreover, in another case, a 54-year-old woman who had RA and type II diabetes experienced periods of severe hypoglycemia hours after receiving etanercept injections [11].

Farrokhi and colleagues [12] describe the case of a 59-year-old woman who had psoriasis and type II diabetes and who experienced symptomatic episodes of hypoglycemia of between 50 to 68 mg/dL six weeks after receiving etanercept injections. This patient exhibited an unexpected improvement in glycemic control shortly after beginning etanercept treatment, despite only minimal change in body weight. Also, a decline of hemoglobin A1C of 2.8% along with a significant decrease of medication requirement for glycemic control was found in this patient within nine months of treatment with etanercept. This finding pointed to a salutary effect on glucose metabolism [12]. In a similar case, a 51-year-old woman who had type II diabetes and who was hospitalized with erythrodermic psoriasis experienced hypoglycemia of as low as 27 mg/dL and convulsions after only seven hours of initiating etanercept, and insulin therapy was discontinued to maintain normal glycemic control [13].

Cheung and Bryer-Ash [14] describe a case of a 72-year-old man who had a history of type II DM and chronic plaque psoriasis and who developed hypoglycemic events of 60 mg/dL or less a few times a week one month after treatment with etanercept. His insulin dose was progressively decreased and then completely discontinued at 16 months of treatment with etanercept, and instead he was treated only with oral anti-diabetic agents. He no longer experienced episodes of hypoglycemia, and at the end of 20 months of treatment with etanercept, his BMI remained unchanged whereas his fasting blood sugar levels improved [14].

In a small pilot study of 18 patients (11 males and seven females), pediatric patients who had newly diagnosed type 1 diabetes and who were treated with etanercept had lower A1C and increased endogenous insulin production, suggesting preservation of beta-cell function. From baseline to week 24, insulin doses decreased 18% in the etanercept group but increased 23% in the placebo group [15].

So far, the only reported cases of hypoglycemia have occurred in individuals who were treated with TNF-α inhibitors and who had a known history of diabetes. In our paper, the presentation is quite unusual since all nine of our patients who developed low blood glucose readings after treatment with a TNF-α inhibitor did not have an underlying history of diabetes. All of our patients were women, whereas cases presented above described both men and women. Whereas all of our cases were asymptomatic, pointing to a more biochemical hypoglycemia, all other reported cases with hypoglycemia occurred in diabetic patients who were treated with TNF-α inhibitors and who were symptomatic. It is possible that TNF inhibitors magnify the clinical effects of hypoglycemia in patients with already-treated diabetes. Additionally, whereas the mean time before development of low blood glucose readings observed in our nine cases was 4.2 months, some instances describe episodes of hypoglycemia occurring within a few hours of TNF inhibitor therapy in patients with pre-existing diabetes.

Our study is a series of cases and aims to serve as a guide for further investigations. However, it carries some limitations. Mainly, it is not a randomized controlled study. The recordings of low blood glucose levels were done retrospectively. Additionally, there might be inadequate exclusion of all other possible causes of low blood glucose readings. Lastly, the threshold for low blood glucose levels used here (< 70 mg/dL) is considered to be higher than the one used in current classic criteria for hypoglycemia, which consists of serum glucose levels of less than 50 to 55 mg/dL and, preferably, documentation of Whipple triad for hypoglycemia (low blood glucose concentration measured with a precise method, presence of symptoms consistent with hypoglycemia, and symptom alleviation upon correction of blood glucose) [16]. Thus, it may be undermining the clinical significance of such low levels of glucose. However, a threshold of blood glucose level of less than 70 mg/dL is often used in anti-diabetic trials to monitor any adverse effects. Also, the neuroendocrine responses begin at an arterial plasma glucose concentration of less than approximately 70 mg/dL [16].

On a more molecular level, the TNF-α molecule appears to inhibit the insulin-mediated uptake of glucose in adipose tissue via a mechanism of downregulating the GLUT-4 receptor. This could lead to a state of insulin resistance [11]. It is possible that, in the patient population presented here, the action of the TNF-α inhibitors brought about increased insulin sensitivity because of the blockade of TNF-α in adipose tissue [17]. Hence, this could be a proposed mechanism for the development of low blood glucose readings observed in our cases.

Hotamisligil and colleagues [18] demonstrated that TNF-α induced serine phosphorylation of insulin receptor substrate 1 (IRS-1), which in turn caused the serine phosphorylation of the insulin receptor. This prevented the normal tyrosine phosphorylation of the insulin receptor and thus interfered with insulin signal transduction. Interleukin-6 and TNF-α have recently been shown [19,20] to induce SOCS-3, a protein that was thought to interfere with cytokine signal transduction but that is now also known to interfere with tyrosine phosphorylation of the insulin receptor and IRS-1 and to cause ubiquitination and proteosomal degradation of IRS-1 [21]. This, in turn, reduces the activation of Akt (protein kinase B), which normally causes the translocation of the insulin-responsive glucose transporter Glut-4 to the plasma membrane. This also induces the phosphorylation of the enzyme nitric oxide synthase and its activation to generate nitric oxide [22]. A newly described protein, TRB3, has also been shown to interfere with the activation of Akt and thus to interfere with the action of insulin [23].

The evidence linking inflammation and DM dates back more than a century. Overproduction of TNF-α has been found in obesity, atherosclerosis, insulin resistance, and type II DM [6]. Also, it was found that inhibition of TNF-α resulted in the improvement of peripheral uptake of glucose in response to insulin in obese mice [6].

One report showed that chronic treatment with infliximab for either RA or psoriatic arthritis brought about a substantial improvement in insulin sensitivity [8]. In the same study, one patient with type II diabetes actually reverted to a state in which glucose tolerance was impaired and insulin therapy ceased. Another study pointed out that glycemic control could improve after 24 months of etanercept therapy [15]. It stands to reason, therefore, that the TNF-α inhibitors used to treat RA could indeed induce a state of hypoglycemia with chronic use.

## Conclusions

In our paper, we presented nine cases of non-diabetic patients who developed episodes of low blood glucose readings after treatment with TNF-α inhibitors. The proposed mechanism might involve induction of increased insulin sensitivity while blocking TNF-α in adipose tissue [17].

This study has some limitations, including the lack of randomization, the use of retrospective recordings, the inadequate exclusion of other possible causes, and the use of a high glucose threshold, undermining the clinical significance of such low levels of glucose. We believe that more investigations are needed to examine this complex relationship between TNF-α and glucose equilibrium.

Physicians using TNF-α inhibitors in the treatment of various rheumatic and autoimmune diseases should be aware of the potential for the development of glycemic disturbance in these patients, a side effect that may be underpublicized. Accordingly, closer monitoring of blood glucose levels should be rendered. Further randomized controlled studies on the relationship between TNF-α inhibitors and glycemic control are warranted, along with more studies examining the possible therapeutic role of TNF-α inhibitors in controlling other autoimmune diseases, such as DM.

## Abbreviations

Akt: protein kinase B; BMI: body mass index; CREST: calcinosis, Raynaud syndrome, esophageal dysmotility, sclerodactyly, and telangiectasia; DM: diabetes mellitus; FMS: fibromyalgia syndrome; IRS-1: insulin receptor substrate 1; RA: rheumatoid arthritis; SLE: systemic lupus erythematosus; SpA: spondyloarthropathy; TNF-α: tumor necrosis factor-alpha.

## Consent

Written informed consent was obtained from the patients for publication of this case report and any accompanying images. A copy of the written consent is available for review by the Editor-in-Chief of this journal.

## Competing interests

The authors declare that they have no competing interests.

## Authors' contributions

JBC analyzed and interpreted patient data regarding hypoglycemic events and was a major contributor in writing the manuscript. BS performed chart review and assisted with the analysis and interpretation of patient data regarding hypoglycemic events. SZ reviewed the case presentation and added to the Discussion section of the manuscript. All authors read and approved the final manuscript.
